# The burden and high prevalence of hypertension in Pakistani adolescents: a meta-analysis of the published studies

**DOI:** 10.1186/s13690-018-0265-5

**Published:** 2018-04-02

**Authors:** Nabi Shah, Qasim Shah, Abdul Jabbar Shah

**Affiliations:** Department of Pharmacy, COMSATS Institute of Information Technology, Abbottabad, 22060 Pakistan

**Keywords:** Hypertension, Meta-analysis, Blood pressure, Prevalence, Pakistan

## Abstract

**Background:**

Hypertension has been recognized as a global health concern for developing countries and is scarcely described in many of these countries. In Pakistan, few population-based surveys evaluated the prevalence of hypertension and there is no current nationally representative study (the latest nationwide survey was conducted more than two decades ago). Objective: The goal of the current study was to estimate the pooled prevalence of hypertension in Pakistani population using meta-analysis approach.

**Methods:**

We searched the published literature using PubMed, Google and Scopus supplemented by a manual search of bibliographies of retrieved articles for population studies providing estimates on the prevalence of hypertension between 1990 and 2017. Studies were included if they defined hypertension as ≥140/90 mmHg and conducted in adults (≥15 years). From the extracted results, the heterogeneity index of the studies was determined using Chi-squared I2 tests and on the basis of heterogeneity, a fixed or random effect model was used to estimates the pooled prevalence of hypertension. Meta-regression was performed to determine those factor of generating heterogeneity.

**Results:**

Of a total of 1240 articles, 18 studies comprising 42,618 participants met the eligibility criteria. The overall pooled prevalence of hypertension was 26.34% (25.93%, 26.75%). Subgroup analysis showed higher urban prevalence 26.61% (21.80%, 31.42%) than the rural dwellers 21.03% (10.18%, 31.87%). The prevalence by decade in 1990s was 19.55% (18.07%, 21.05%), in 2000s 23.95% (16.60%, 31.30%) and in 2010s 29.95% (24.13%, 35.77%). Similarly, the pooled prevalence was 24.99% (19.70%, 30.28%) in males and 24.76% (16.76%, 32.76%) in females. We recorded high burden of hypertension among the adult Pakistanis when compared to the data published in local and international journals 23.32% (18.9%, 27.74%) and 27.44% (20.97%, 33.91%). We also found differences in the prevalence of hypertension among small, medium and large studies.

**Conclusion:**

Comparing data from previous studies in Pakistan, we found a higher prevalence in urban areas and among males. The prevalence over time is likely to increase faster, further our results underscore the importance of good quality long-term studies that will help to understand hypertension better and implement effective prevention and management programs.

## Introduction

Hypertension is a modifiable and major risk factor for coronary artery disease, heart failure, cerebrovascular disease and chronic renal failure [1, 2]. It is also recognized as a global chronic, non-communicable disease and a “silent killer” due to its high mortality rates and lack of early symptoms. One-quarter of the world’s adult population is hypertensive and it is estimated that by 2025 this figure is likely to increase to 29% [3]. The high prevalence of hypertension in both developed and developing countries makes it a significant factor for mortality and morbidity [4]. Unfortunately, the prevalence of hypertension is growing rapidly in developing countries which are undergoing epidemiological transitions, economic improvement, urbanization and longer life expectancy [5, 6]. Adequate management of hypertension can effectively reduce the risks of stroke, myocardial infarction [4], chronic kidney disease [7] and heart failure [8].

Although hypertension has been recognized as a major risk factor for cardiovascular morbidity and mortality worldwide, there is a lack of nationwide prevalence data in most of the developing countries [5, 9]. Such information is needed in order to determine the economic burden of hypertension, as well as to optimize health resources allocation towards improvement for its detection, treatment and control. In Pakistan, many population-based surveys, representative of cities and of one province, have been conducted in the past few decades. However, there are no estimates of the prevalence of hypertension for the whole country or of trends in the past decades. The National Health Survey of Pakistan (NHSP) estimated that nearly 18.9% of the Pakistan people aged 15 years and older were hypertensives [10, 11].

Pakistan has a high rate of urbanization, where individuals consume a diet high in salt, calories, saturated fat and low in fruits and vegetables. Several studies hypothesized that these changes to contribute to a higher prevalence of hypertension in urban than rural populations [12, 13]. Pakistan needs to improve strategies for the prevention of hypertension which needs a sensible plan of action for prevention and improvement of current policies against hypertension. Although we found many small to intermediate-scale studies from around the country that estimated the prevalence of hypertension, there is no nationwide study on the prevalence of hypertension; the latest nationwide investigation is now more than 20 years old [10, 11]. Therefore, we performed the current meta-analysis to systemically review the findings of all current studies and estimate the overall prevalence of hypertension in the Pakistani population.

## Methods

### Literature search strategy

We searched published articles for epidemiologic studies on the prevalence of hypertension by conducting electronic database searches using PubMed, Google Scholar and Scopus. The search was conducted using three blocks of concepts; the first block with terms of hypertension (“hypertension”[Mesh] or “blood pressure”[Mesh] or “hypertension” or “blood pressure”), the second block with terms related to Pakistan (“Pakistan” or “Pakistani” or “Pakistani population”) and the third block with terms (“prevalence” or “epidemiology study” or “epidemiologic studies” or “epidemiology” or “statistics”, or “numerical data” or “prevalence”). MeSH term was only applied to the articles retrieved from PubMed. Similarly, no search limits were applied either to study design or sample size. Independent manual search on reference lists of the retrieved articles was also performed. A flowchart of information pertaining to identification, screening, eligibility, and final studies included was constructed according to “Preferred Reporting Items for Systematic Reviews and Meta-analyses” (PRISMA) statements [14].

We included all the epidemiologic studies which reported the overall prevalence of hypertension. Studies that used random sampling or census data to find individuals with hypertension among adult population (≥15 years old) and between 1990 and August 2017. Similarly, all source studies included were original research work and contain the minimum information necessary to calculate pooled analysis of prevalence (number of subjects and number of hypertension events). Hypertension prevalence was the main summary measure used in the current study. Therefore, to satisfy the analysis requirements and reduce selection deviation, the studies were included if they explicitly defined hypertension as average systolic BP ≥ 140 mmHg and diastolic BP ≥ 90 mmHg, or self-reported current treatment for hypertension with an antihypertensive medication as defined by the Seventh Annual Report of the Joint National Committee (JNC-7) guidelines [15].

Assessment of the study quality was carried out by analyzing the characteristics of the studies about the design (size and type of sample), the possibility of misclassification of outcome (number of measures, measures used for hypertension classification, treatment of discrepant measures) and overall estimates by gender prevalence. We excluded studies if the participants were limited to a particular occupation, population and age group. Studies were also excluded if they utilized nonrandom sampling or non-JNC-7 standards (e.g. self-reported hypertension without measuring blood pressure). Similarly, we also excluded letters, abstracts, conference proceedings and reviews.

### Study selection

The first screening comprised a double-screening of titles and abstracts. Results those met explicit inclusion criteria were included. To avoid bias in the data from the articles, two authors independently collected the data and compared results, with any disagreements about the selection being resolved through consensus or consultation with a third author. Full-text articles for the selected title were retrieved. Reference lists of the retrieved articles were searched for additional publications. The retrieved studies were assessed again by two independent authors to ensure that they satisfied the inclusion criteria. When there was more than one publication with data from the same study the most comprehensive article was selected.

### Data extraction

All searched articles from the electronic database were combined using Endnote and duplicates were removed. A form was drawn up to extract data from the full text of the articles, and besides hypertension prevalence, other relevant information was also extracted by two investigators, cross-checked and resolved for disagreements by consensus. For all included studies, the following information was collected: year of publication, year of investigation, city, sampling method, data collection method, diagnostic criteria of hypertension, the age of the subject, number of total individuals in the study, number of individuals with hypertension, gender distribution and age-specific prevalence. Studies were also included if they did not describe the technique of BP measurement but made reference to a guideline. Authors were contacted to obtain full texts or estimates not present in the articles.

### Statistical analysis

The standard error (SE) of prevalence and 95% confidence interval (95% CI) was calculated from the reported percentage prevalence and sample size for each of the studies. SE was calculated manually as “ROOT p x (1-p)/n and 95% CI was calculated as p ± SE x 1.96”. Where p is the proportion of prevalence and n is the reported sample size. We used 95% confidence interval (CI) to gauge the precision of the summary estimates. Overall heterogeneity was assessed by reporting the *I*^*2*^ (% residual variation due to heterogeneity) for each of the pooled estimates [16]. As the difference between the trials was very large (more than 90% inconsistency), a random effect model was used to pool the prevalence of hypertension. In order to deal with heterogeneity, a secondary analysis was necessary to perform subgroup analysis. Subgroups were defined gender, geographical or regional differences (urban and rural), year range of investigation, publication type (local and international) and study size (n = < 1000 small, n = < 4000 medium and n= > 4000 large). Publication bias was evaluated by testing for funnel plot asymmetry, Begg’s Test and Egger’s regression test. The suspected factors of heterogeneity were examined using meta-regression. Prevalence was compared using t-test and the Chi-square test, level of significance was set at 0.05. All analyses were performed using STATA version 11.2 (Stata Crop., College Station, Taxas, USA).

## Results

### Study selection

**Fig. 1:**
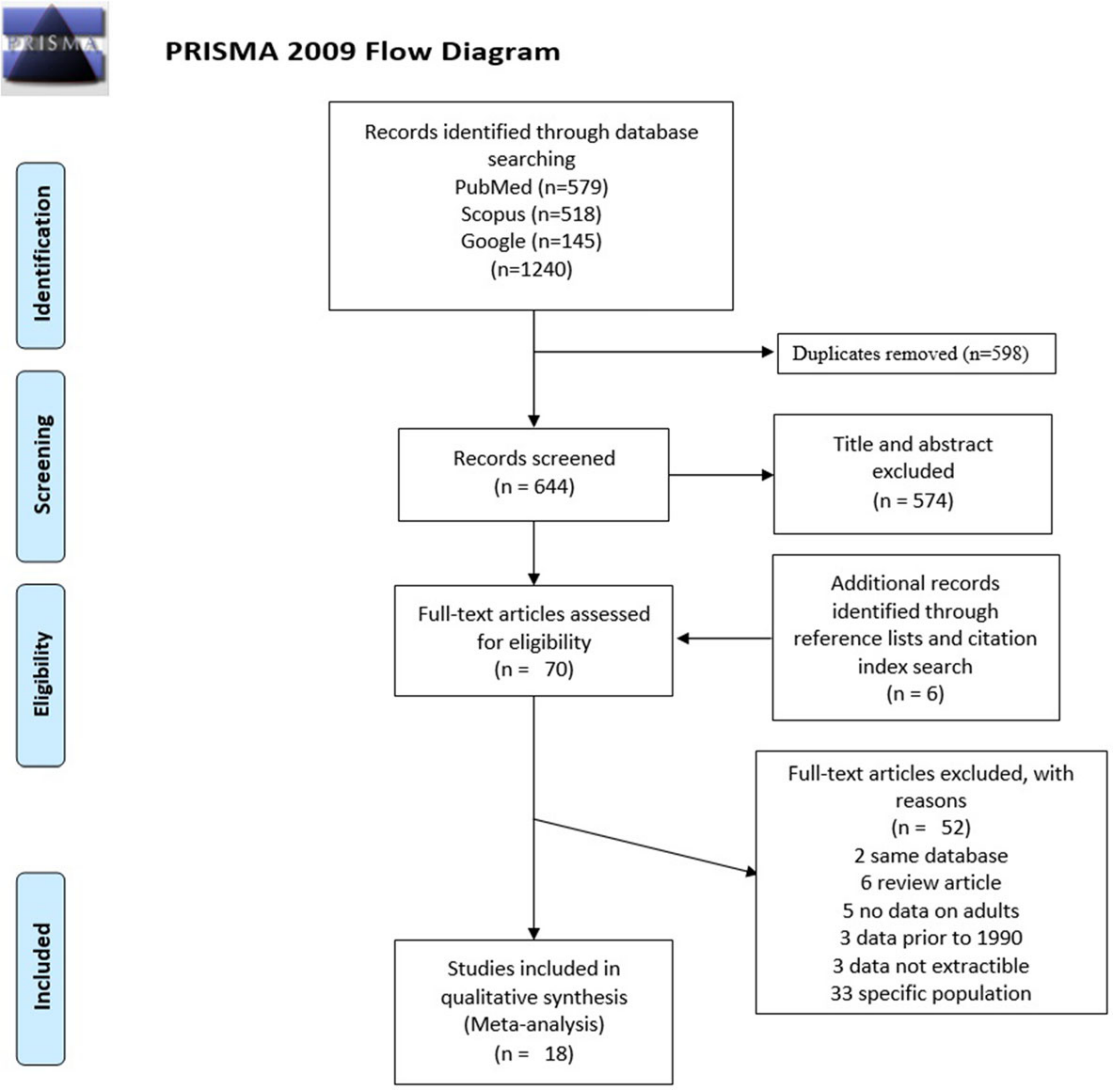
Flow diagram of the study selection process. As shown our initial searches resulted in 1240 citations. After screening title and abstracts, 70 studies were considered potentially eligible and retrieved in full text, of these 18 studies were subsequently included in the meta-analysis

From the initial search, 1240 references were identified in the electronic database. In the first screening 598 duplicates were removed and in the second screening, 574 abstracts were screened by the inclusion criteria, as described in the methods. Full-text assessment of 70 potentially relevant studies resulted in total 18 eligible studies to include in the final meta-analysis [11, 12, 17–32]. These 18 studies contained a total of 42,618 individuals determined to be suitable for meta-analysis. Flowchart of the studies selection process is shown in Fig. 1 and detailed information of data extracted from these studies is compiled in Table 1. Of these 18 studies, one study [19] did not mention age criteria, twelve studies [11, 18, 20–27, 30, 32] limited to 15 or above while five studies [12, 17, 28, 29, 31] was limited to 25 and above. Similarly, the range of frequency of BP measurements varied from 2 to 3 and the intervals between each measurement ranged between 5 to 30 min. Two studies [24, 26] did not mention the BP measuring frequency, seven studies [11, 12, 17, 20, 23, 25, 32] took two BP measurements while nine studies [18, 19, 21, 22, 25, 28–31] used an average of three BP readings. Moreover, the sample size varied considerably among studies with a lower sample size of 151 [17] and higher sample size of 13,722 [32]. Most of the studies measured blood pressure either with aneroid or mercury manometer.

**Table 1:** Characteristics of the primary studies included in the meta-analysis

Sr. no	Author name (year)	Year conducted	Criteria	BP measurement frequency	Population	Place of study	Age	Sample size (n)			Prevalence of hypertension (%)		
								Total	Male	Female	Total	Male	Female
1	Akatsu and Aslam (1996)	1996	≥140/90	2	Urban	Karachi	> 25	151	–	151	16.55	–	16.55
2	Shah SM, et al. (2001)	2001	≥140/90	3	Rural	GB	> 18	4203	1406	2797	14.89	13.72	15.48
3	Rafique G, et al. (2003)	2003	≥140/90	3	Urban	Karachi	NR	264	–	–	23.86	–	–
4	Dodani S, et al. (2004)	2004	≥140/90	2	Urban	Karachi	> 18	1137	–	–	24.71	–	–
5	Iqbal SP, et al. (2004)	2004	≥140/90	3	Urban	Karachi	> 18	370	294	76	18.91	16.66	27.63
6	Safdar S, et al. (2004)	2002	≥140/90	3	Urban	Karachi	> 18	857	172	685	26.02	34.3	23.94
7	Aziz KU, et al. (2005)	2005	≥140/90	2	Urban	Karachi	> 18	946	476	470	23.25	23.11	23.4
8	Siddique H, et al. (2005)	2005	≥140/90	NR	Urban	Karachi	> 15	198	63	135	15.15	17.46	14.07
9	Tazeen H. Jafar, et al. (2003)	1990–1994	≥140/90	3	Both	Pakistan	> 15	9442	4409	5033	19.75	21.75	18
10	Tareen MF, et al. (2011)	2006–2009	≥140/90	2	both	Punjab	30–75	2495	1271	1224	24.21	28.16	20.42
11	Alam HM, et al. (2013)	2010–2011	≥140/90	2	Urban	Karachi	> 20	176	–	–	23.86	–	–
12	Khan FS, et al. (2013)	2010–2011	≥140/90	NR	Urban	Karachi	> 15	500	–	–	18	–	–
13	Aslam A, et al. (2014)	2010–2011	≥140/90	3	Urban	Karachi	> 15	554	–	–	26.35	–	–
14	Jessani S, et al. (2014)	2004–2005	≥140/90	3	Urban	Karachi	> 40	2873	–	–	44.1	–	–
15	Irzola VE, et al. (2016)	2010–2011	≥140/90	3	Urban	Pakistan	> 35	2584	1213	1371	42.29	38.16	45.95
16	Zafar J, et al. (2016)	2014	≥140/90	3	Urban	Rawalpindi	> 18	404	–	–	38.36	–	–
17	Gupta R, et al. (2017)	2017	≥140/90	3	Urban	Karachi	35–70	1742	–	–	24.91	–	–
18	Shafi ST, et al. (2017)	2008–2015	≥140/90	2	Rural	Punjab	≥ 18	13,722	8366	5356	35.06	30.81	41.71

**Fig. 2:**
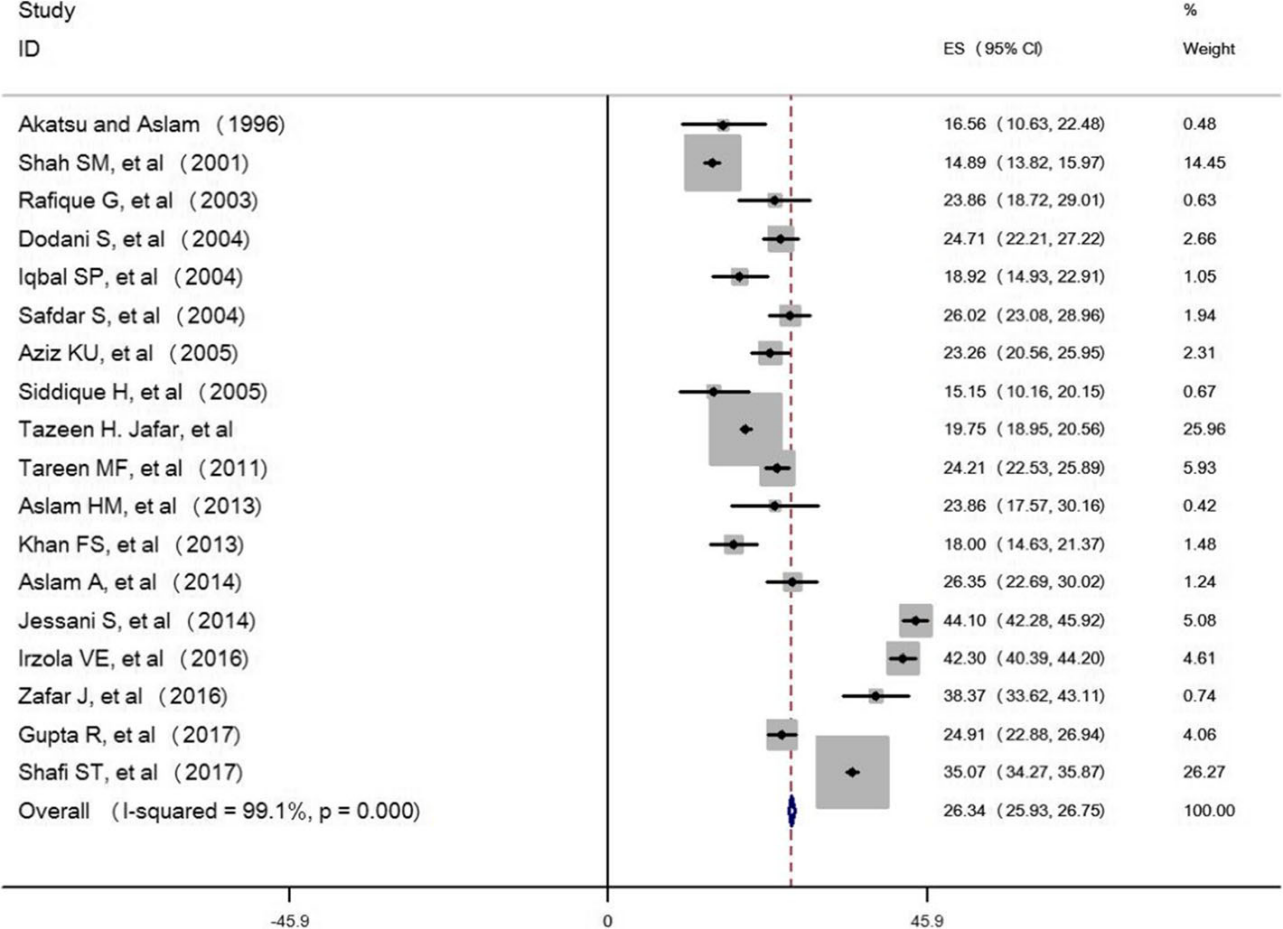
Forest plot of all selected studies shows prevalence estimates (boxes) with 95% confidence intervals (bar) for each study selected; pooled prevalence estimates are represented as diamonds in this plot

Pooled prevalence estimates for 42,618 individuals in the 18 final studies are described according to the criteria used for defining hypertension. The overall prevalence of hypertension was found to be 26.34% (25.93%, 26.75%) shown in Fig. 2, using heterogeneity test results, considerable heterogeneity was found (*I*^*2*^ = 99.1%, *p* < 0.001). Therefore, the overall prevalence was estimated using random effect model 25.64% (20.97%, 30.30%).

Table 2 demonstrates the pooled prevalence of hypertension in all sub-groups stratified by gender, geographical region, study year, type of publication (local vs international) and study size. The pooled prevalence of hypertension among males and females was 24.99% (19.70%, 30.28%) and 24.76% (16.76%, 32.76%), respectively. Heterogeneity was significantly present in both males and females (I^2^ = 98.3%; *p* < 0.001) and (I^2^ = 99.3%; *p* < 0.001), respectively. The t-test showed a significant difference in the prevalence of hypertension between males and females (*p* = 0.0118).

**Table 2:** Hypertension prevalence according to gender, geographical region, study year, type of publication and study size

Study or Subgroup	No. of studies	Prevalence (95% CI) (%)	N	Heterogeneity test		Publication bias test	
				I^2^ (%)	*P*	*P* (Begg’s test)	*P* (Egger’s test)
Fixed Effect Model	18	26.34 (25.93, 26.75)	42,618	99.1	< 0.001	0.602	0.294
Random Effect Model	18	25.64 (20.97, 30.30)	42,618	99.1	< 0.001	0.602	0.294
Sex (Male)	9	24.99 (19.70, 30.28)	17,670	98.3	< 0.001	0.835	0.752
Sex (Female)	10	24.76 (16.76, 32.76)	17,298	99.3	< 0.001	0.421	0.858
Urban	16	26.61 (21.80, 31.42)	17,224	98.4	< 0.001	0.787	0.308
Rural	4	21.03 (10.18, 31.87)	25,394	99.8	< 0.001	0.497	0.393
Study years (1990s)	2	19.55 (18.07, 21.05)	9593	38.9	0.201	0.317	–
Study years (2000s)	9	23.95 (16.60, 31.30)	13,343	98.9	< 0.001	0.532	0.57
Study years (2010s)	7	29.95 (24.13, 35.77)	19,682	97.8	< 0.001	0.652	0.325
Local journal	8	23.32 (18.9, 27.74)	3366	88.6	< 0.001	0.805	0.762
International journal	10	27.44 (20.97, 33.91)	39,252	99.5	< 0.001	0.929	0.755
Small Studies	10	23.27 (22.03, 24.50)	10,831	87.9	< 0.001	0.788	0.855
Medium Studies	5	32.65 (31.79, 33.52)	4420	99.1	< 0.001	1	0.737
Large studies	3	24.73 (24.23, 25.23)	27,367	99.8	< 0.001	0.117	0.573

*Abbreviation*: *N* total number of subjects from the included studies

The pooled prevalence of hypertension in the population living in the urban area 26.61% (21.80%, 31.42%) 26.45% (21.4%, 31.46%) was higher than was observed in the rural area 21.03% (10.18%, 31.87%), Heterogeneity was observed between urban and rural studies (I^2^ = 98.4%; *p* < 0.001 and I2 = 99.8%; p < 0.001), respectively. We found significant difference in the prevalence of hypertension among the two geographical regions (*p* < 0.001).

The pooled prevalence of hypertension increased with time, and was 19.55% (18.07%, 21.05%) during 1990–1999, increasing to 23.95% (16.60%, 31.30%) during 2000–2009 and 29.95% (24.13%, 35.77%) during 2010–2017. We noted significant heterogeneity in the studies conducted during 2000s (I^2^ = 98.9%; *p* < 0.001) and 2010s (I^2^ = 97.8%; *p* < 0.001), while consistency in the prevalence during 1990s (I^2^ = 38.8%; *p* 0.201). We found significant differences in subgroup prevalence of hypertension with time (*p* < 0.001).

Similarly, the prevalence of hypertension was 23.32% (18.90%, 27.74%) in data published in local journals and 27.44% (20.97%, 33.91%) in data published in international journals. Moreover, significant heterogeneity was observed between local and international journals ((I^2^ = 88.6%; *p* < 0.001 and I^2^ = 99.5%; *p* < 0.001), respectively. The difference in the prevalence of hypertension between local and international journals was significantly different (*p* < 0.001).

Furthermore, we found the pooled prevalence of hypertension increased by the size of study, the prevalence in small studies was 23.27% (22.03%, 24.50%), than medium studies 32.65% (31.79%, 33.52%) and large studies 24.73% (24.23%, 25.23%). Significant heterogeneity was observed among small, medium and large studies (I^2^ = 87.9%; *p* < 0.001 and I^2^ = 99.1%; *p* < 0.001 and I^2^ = 99.8%; *p* < 0.001, respectively). Subgroup prevalence of hypertension was found significantly different among the groups (*p* < 0001).

**Fig. 3:**
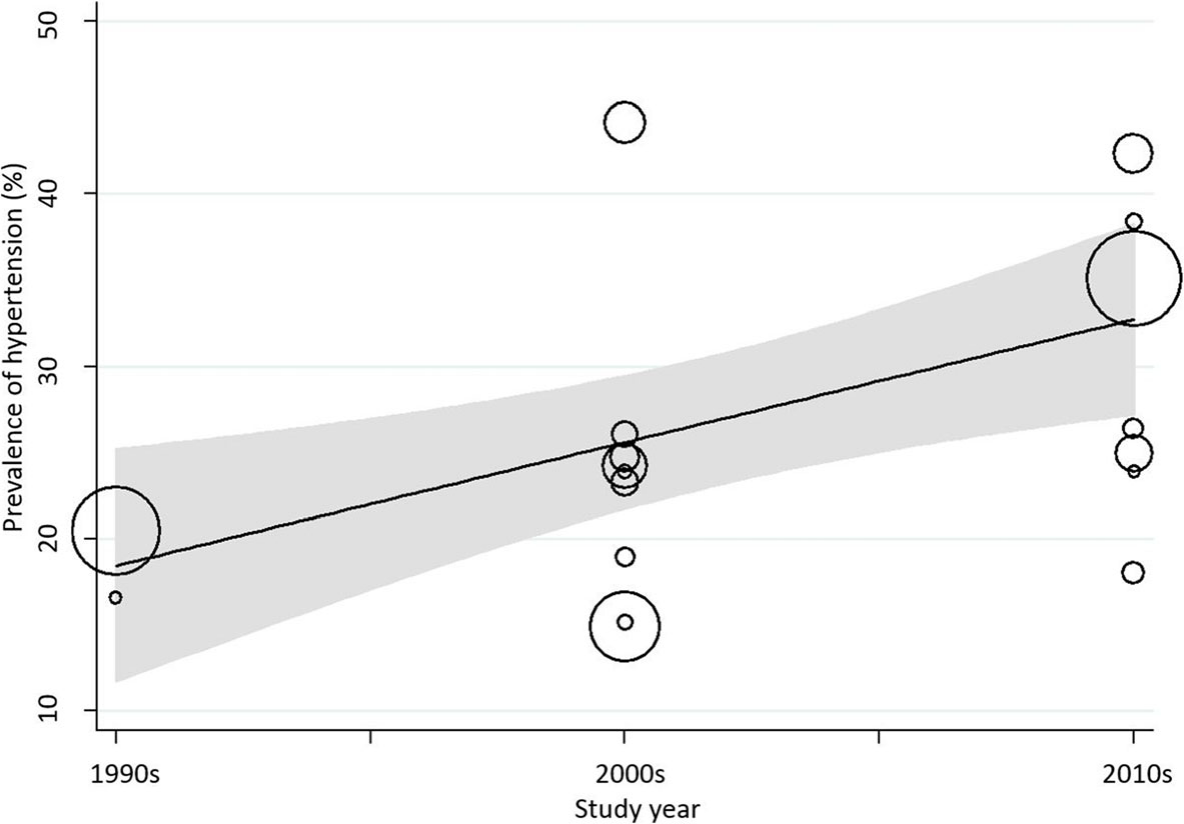
Meta-regression of hypertension prevalence against study year

**Fig. 4:**
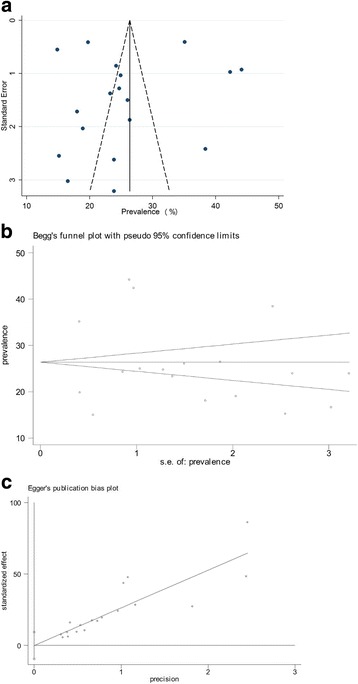
**a** Funnel plot detailing publication bias in the studies reporting the prevalence of hypertension in Pakistan. **b** Begg’s regression test of the overall prevalence of hypertension. **c** Egger’s regression test of the overall prevalence of hypertension

A high level of heterogeneity between studies and subgroups was observed, therefore we then performed meta-regression analyses to identify the source of heterogeneity associated with the overall prevalence of hypertension. Variable included in the analyses were gender, geographical region, study year, publication type and study size. The results of meta-regression analyses showed that only study year (coefficient + 5.87 [95% CI -0.521 to 12.27], *p* 0.062) (Fig. 3) accounted for marginal significant heterogeneity in the prevalence of hypertension between studies. Table 3 shows a list of possible factors that could determine the observed heterogeneity in hypertension prevalence in the current analyses. Similarly, the funnel plot the study standard error by prevalence was asymmetric (Fig. 4a), suggesting publication bias, however, this was not confirmed either by Begg’s test or Egger’s test shown in Table 2 and Fig. 4b and c.

**Table 3:** Results of meta-regression for the prevalence of hypertension in Pakistan

Covariate	Meta-regression coefficient	95% confidence interval	*P* value	Variance explained %
Gender ratio (male vs female)	−0.1773606	−9.89355, 9.538831	0.97	−6.00
Geographical region (rural vs urban)	5.592422	−4.85746, 16.04	0.276	1.67
Study year (continuous)	0.585197	−0.03395, 1.204341	0.062	15.21
Study year (1990 vs 2000 vs 2010)	5.877195	−0.52148, 12.28	0.069	14.34
Publication type (local vs international)	4.161891	−4.86441, 13.18819	0.343	−0.31
Sample size (small vs medium vs large)	1.713683	−4.27034, 7.697709	0.552	−4.20
Sample size (continuous)	0.0005039	−0.00076, 0.00177	0.411	−2.02

## Discussion

Hypertension is an important public health problem in both the economically developed and developing world. In this comprehensive systemic review, we described estimates of the prevalence of hypertension in the adult Pakistani population. At present, there is lack of nationwide data regarding hypertension prevalence. Domestic and international literature searches found only one recent review, which focused on hypertension in Asian countries [33]. Therefore, the present meta-analysis is relevant to the current healthcare need and based on a large number of participants. This meta-analysis provided a reliable estimate of the prevalence of hypertension in the Pakistani population. Our results present a detailed view of the overall prevalence and burden of hypertension by gender, geographical region and estimate of hypertension prevalence with time, comparison of the overall prevalence of hypertension published in local and international journals and by study size.

Our results indicated over 2.86 (around 3) in 10 of residents older than 15 years is hypertensive. The majority of the studies were conducted in urban Pakistan and a few studies estimated the prevalence of hypertension in the rural areas. Rural areas have less pooled prevalence of hypertension as compared to urban areas, which strongly implicates differences in lifestyle as an explanatory factor. The higher level of obesity and increased salt and fat intake from consuming more processed foods and engaging in jobs with minimal physical activity are a most likely explanation for the higher prevalence of hypertension in the urban population. Prevalence of hypertension has been on the rise in the recent decades, national sampling study from 1990 to 1994, reported the prevalence to be 19.75% [10, 11, 34], this was lower from the current estimate of hypertension prevalence 26.73%. Although varying methodologies make comparisons complex, the values from the current study are higher than the previous national health survey. The pooled prevalence of hypertension having increasing trend with the passage of time among studies performed during the 1990s than in the 2000s and 2010s. The increase in hypertension prevalence could be possibly explained by economic development, urbanization, ageing, lifestyle changes, changing in eating habits and environmental pollution with time. Similarly, it may also be attributed to the increase in overweight and obese individuals, smoking and high salt diet. It has been reported that the percentage of the Pakistani population living in urban areas has increased from 17.6% in 1951 to 35.2% in 2013 [35]. Given this trend, the urban proportion is likely to continue to increase. Therefore, effective treatment and prevention measures focusing on the high-risk urban population will have a profound and favourable impact on public health. Moreover, due to heterogeneity in the studies, a national epidemiological study should be conducted to confirm if the prevalence of hypertension is truly increasing.

We found the pooled prevalence of hypertension in females to be slightly greater than males. The greater prevalence of hypertension in females has also been shown in many other studies [36, 37]. Our results are contrary to the data reported previously [38]. Women were reported to have better detection, treatment and control rates than men in high-income countries, due to pregnant women have more opportunities to have their blood pressure checked. However, because of data limitations and the risk of ecological fallacy we could not assess the reasons for such a contradiction in the Pakistani females. However, greater body mass index (BMI) among females and their lifestyle were counting as a possible factor in other studies [39]. Oral contraceptives and menopause can be other factors affecting the prevalence of hypertension in females [40, 41]. Other possible explanation could be that, in Pakistan where resources for health are generally limited and individuals and households usually have to pay for the utilization of services and treatment, it is very likely that health conditions such as hypertension will receive little attention.

We found significant heterogeneity in the current analyses, which need caution in extrapolating the results, though we performed meta-regression, we were unable to explain the causes of heterogeneity. The age range of the included studies varied and could provide a guide to the comparability of the studies. However, in most studies data were reported by the overall prevalence of hypertension across the population, with a few were reported by gender and a very few studies reported by age groups. It has been shown that the distribution of hypertension is influenced by a number of factors such as by age, gender and racial composition. Similarly, studies with small sample size and publications type could have an influence on this difference, which may have contributed to some bias in the results. Besides methodological causes, the high heterogeneity of the results may also be due to natural differences among the subjects included in the studies, since Pakistani cities are socioeconomically different from each other. The current pooled analysis of the prevalence of hypertension is an attempt to fill the lack of national data. However, the estimates of the prevalence of hypertension do not adequately represent all the major Pakistani cities. The key strength of the current meta-analysis is that it is the first comprehensive analysis on the prevalence of hypertension from Pakistan. Second, this meta-analysis focused on the most recent literature included to explore the progression of the overall prevalence of hypertension and in different subgroups. While we tried our best to adhere to the guidelines for reporting meta-analysis including 18 studies with large pooled sample size, still our study had several limitations that merit attention.

Although we performed a meta-regression, our study was only designed to report the pooled prevalence of hypertension in the general population and subgroups. Heterogeneity of prevalence was found high in the current analysis, therefore, we assumed as such, that there were other factors more likely contributing to heterogeneity, including genetic factors, environmental factors, tobacco use, physical inactivity and high salt intake [42, 43]. Similarly, strict inclusion and exclusion criteria were used to identify studies in the literature, both inter and intra-study measurement error in the ascertainment of blood pressure, classification of subjects and other indices will have occurred. For example, the number of blood pressure measurements could influence the classification of patients as hypertensive [44]. Moreover, the investigation bias may also affect the meta-analysis outcomes, because there were great disparities in the distribution of healthcare resources between cities and rural areas in Pakistan. And lastly, we did not include data from the participants living outside Pakistan.

## Conclusion

This large systemic review revealed the changing trends in the prevalence of hypertension in Pakistan. We observed higher prevalence rates in urban population compared to the rural population. An increasing trend was found in urban and rural population and also in both genders. Moreover, a considerable increase was found in the prevalence of hypertension over time and further, we stress over the need for good quality studies focusing on hypertension and its treatment in Pakistanis for hypertension management. In summary, the results of our study are not a substitute for a nationwide prevalence study, therefore, one should be careful in prescribing direct policy recommendations from this meta-analysis alone. However, still our findings do reveal an important current healthcare issue and our results summarized better estimates available that can serve as a reference for public health policy.
